# Different clinical characteristics and impact of carbapenem-resistance on outcomes between *Acinetobacter baumannii* and *Pseudomonas aeruginosa* bacteraemia: a prospective observational study

**DOI:** 10.1038/s41598-022-12482-0

**Published:** 2022-05-20

**Authors:** Chan Mi Lee, Young-Jun Kim, Sook-In Jung, Seong Eun Kim, Wan Beom Park, Pyoeng Gyun Choe, Eu Suk Kim, Chung-Jong Kim, Hee Jung Choi, Shinwon Lee, Sun Hee Lee, Younghee Jung, Ji Hwan Bang, Shinhye Cheon, Yee Gyung Kwak, Yu Min Kang, Kyung-Hwa Park, Kyoung-Ho Song, Hong Bin Kim, The Korea INfectious Diseases (KIND) Study Group, Chan Mi Lee, Young-Jun Kim, Sook-In Jung, Seong Eun Kim, Wan Beom Park, Pyoeng Gyun Choe, Eu Suk Kim, Chung-Jong Kim, Hee Jung Choi, Shinwon Lee, Sun Hee Lee, Younghee Jung, Ji Hwan Bang, Shinhye Cheon, Yee Gyung Kwak, Yu Min Kang, Kyung-Hwa Park, Kyoung-Ho Song, Hong Bin Kim

**Affiliations:** 1 Department of Internal Medicine, Seoul National University Bundang Hospital, Seoul National University College of Medicine, Seongnam, Republic of Korea; 2 Department of Internal Medicine, Wonkwang University Hospital, Iksan, Republic of Korea; 3 Department of Infectious Diseases, Chonnam National University Medical School, Gwangju, Republic of Korea; 4 Department of Internal Medicine, Seoul National University Hospital, Seoul National University College of Medicine, Seoul, Republic of Korea; 5 Department of Internal Medicine, Ewha Womans University Hospital, Seoul, Republic of Korea; 6 Department of Internal Medicine, Pusan National University Hospital, Busan, Republic of Korea; 7 Department of Internal Medicine, Hallym University Sacred Heart Hospital, Anyang, Republic of Korea; 8 Department of Internal Medicine, Seoul Metropolitan Boramae Hospital, Seoul, Republic of Korea; 9 Department of Internal Medicine, Chungnam National University Hospital, Daejeon, Republic of Korea; 10 Department of Internal Medicine, Inje University Ilsan Paik Hospital, Goyang, Republic of Korea; 11 Department of Internal Medicine, Kangwon National University Hospital, Chuncheon, Republic of Korea; 12 Present Address: Department of Internal Medicine, Myongji Hospital, Goyang, Republic of Korea

**Keywords:** Bacteria, Pathogens

## Abstract

This study aimed to evaluate the differences in clinical characteristics and impact of carbapenem resistance (CR) on outcomes between *Acinetobacter baumannii* (Ab) and *Pseudomonas aeruginosa* (Pa) bacteraemia. We prospectively identified all patients with Ab and Pa bacteraemia in 10 hospitals over 1 year. Treatment failure was defined as all-cause 30-day mortality, persistent bacteraemia, or recurrent bacteraemia within 30 days. We included 304 Ab and 241 Pa bacteraemia cases. CR was detected in 216 patients (71%) with Ab bacteraemia and 55 patients (23%) with Pa bacteraemia. Treatment failure was significantly higher in CR-Ab than in CR-Pa (60.6% vs. 34.5%, *P* = 0.001). In Ab, severe sepsis or septic shock and high Pitt bacteraemia score were independent risk factors for treatment failure in the inappropriate empirical antibiotics group. In Pa, hospital-acquired infection and high Pitt bacteraemia score were independent risk factors for treatment failure in both groups. CR was an independent risk factor in Ab for treatment failure in both groups, but not in Pa bacteraemia. We demonstrated significant differences in clinical characteristics and impact of CR on clinical outcomes between Ab and Pa bacteraemia, suggesting that different treatment approaches may be needed.

## Introduction

Antimicrobial resistance is a major threat to public health. A recent report from the Centers for Disease Control and Prevention concluded that the threat of antibiotic resistance was greater than previously understood, and listed carbapenem resistance in non-fermenting gram-negative bacteria, such as *Acinetobacter baumannii* (Ab) and *Pseudomonas aeruginosa* (Pa), as urgent threats^1^. Various resistance mechanisms contribute to carbapenem resistance in Pa, including changes in porin expression, upregulation of efflux pumps, and production of carbapenemase. On the other hand, the mechanism of carbapenem resistance in Ab is more likely due to carbapenemase-mediated mechanisms. The Oxa-23 carbapenemase is the most common carbapenemase in Ab^2^. There are limited treatment options for carbapenem resistance; due to its severity and poor clinical outcomes, it is an emerging public health concern^3–5^. Therefore, to improve the treatment of antibiotic-resistant infections, identifying their clinical features is critical.

Both carbapenem-resistant Ab (CR-Ab) and carbapenem-resistant Pa (CR-Pa) are important causes of healthcare-associated infections^6^, but they have different antibiotic susceptibility and clinical features. However, limited information is available on the clinical impact of carbapenem resistance in Ab and Pa. Due to the limited treatment options for CR-Ab and CR-Pa, it is important to elucidate the impact of carbapenem resistance on the clinical outcomes and risk factors for treatment failure. Therefore, we aimed to compare the clinical characteristics and impact of carbapenem resistance on clinical outcomes of Ab and Pa bacteraemia. By determining the impact of carbapenem resistance and risk factors associated with the clinical outcomes of Ab and Pa, we intend to contribute to knowledge regarding the treatment of antibiotic-resistant infections.

## Results

### Clinical characteristics of Ab and Pa bacteraemia

A total of 545 cases of Ab or Pa bacteraemia were identified during the 1-year study period, of which 304 were Ab bacteraemia and 241 were Pa bacteraemia. Of the 304 Ab bacteraemia cases, 216 (71%) were CR-Ab and 88 (29%) were carbapenem-susceptible Ab (CS-Ab). Of the 241 Pa bacteraemia cases, 55 (23%) were CR-Pa and 186 (77%) were carbapenem-susceptible Pa (CS-Pa).

The clinical characteristics of Ab bacteraemia according to carbapenem susceptibility are listed in Supplementary Table S1. Catheter-related bloodstream infection and pneumonia were more frequent (42.1% vs. 28.4%, *P* = 0.026; 36.1% vs. 9.1%, *P* < 0.001); severe sepsis or septic shock (53.2% vs. 21.6%, *P* < 0.001) and inappropriate empirical therapy (75.0% vs. 19.3%, *P* < 0.001) showed higher percentages; and all-cause 30-day mortality and treatment failure rates were significantly higher (57.5% vs. 15.5%, *P* < 0.001; 60.6% vs. 15.9%, *P* < 0.001) in the CR-Ab group than in the CS-Ab group.

The clinical characteristics of Pa bacteraemia according to carbapenem susceptibility are described in Supplementary Table S2. The CR-Pa group showed significantly higher percentages of hospital-acquired infection (HAI; 61.8% vs. 45.2%, *P* = 0.030); underlying liver disease (25.5% vs. 14.0%, *P* = 0.044); and inappropriate empirical therapy (52.7% vs. 26.3%, *P* < 0.001) than the CS-Pa group. There was no significant difference in treatment failure between the CR-Pa and CS-Pa groups (34.5% vs. 24.7%, *P* = 0.150).

The clinical characteristics of the CR-Ab and CR-Pa groups are compared in Table 1. The CR-Ab group showed significantly higher percentages of severe sepsis or septic shock and inappropriate empirical therapy, and all-cause 30-day mortality and treatment failure were significantly higher than in the CR-Pa group.

**Table 1:** Clinical characteristics of patients with carbapenem-resistant *Acinetobacter baumannii* (CR-Ab) and carbapenem-resistant *Pseudomonas aeruginosa* (CR-Pa) bacteraemia.

Variables	Total (n = 271)	CR-Ab (n = 216)	CR-Pa (n = 55)	*P*
Age, mean (± SD)	65.6 (± 16.9)	66.0 (± 17.2)	64.1 (± 15.4)	0.469
Male	177 (65.3)	143 (66.2)	34 (61.8)	0.542
ICU stay at bacteraemia onset	139 (51.3)	127 (58.8)	12 (21.8)	** < 0.001**
Hospital-acquired infection	218 (80.4)	184 (85.2)	34 (61.8)	** < 0.001**
Healthcare-associated infection	260 (95.9)	211 (97.7)	49 (89.1)	**0.004**
Mixed bacteraemia	29 (10.7)	18 (8.3)	11 (20.0)	**0.012**
**Site of infection**				
Primary bacteraemia	76 (28.0)	58 (26.9)	18 (32.7)	0.387
CRBSI	103 (38.0)	91 (42.1)	12 (21.8)	**0.006**
Pneumonia	82 (30.3)	78 (36.1)	4 (7.3)	** < 0.001**
Urinary tract infection	55 (20.3)	45 (20.8)	10 (18.2)	0.662
Intra-abdominal infection	26 (9.6)	16 (7.4)	10 (18.2)	**0.015**
**Underlying medical condition**				
Charlson’s WIC ≥ 3	216 (79.7)	172 (79.6)	44 (80.0)	0.951
Heart disease	28 (10.3)	24 (11.1)	4 (7.3)	0.404
Lung disease	27 (10.0)	26 (12.0)	1 (1.8)	**0.024**
Chronic kidney disease	51 (18.8)	43 (19.9)	8 (14.5)	0.364
Liver disease	56 (20.7)	42 (19.4)	14 (25.5)	0.326
Diabetes mellitus	79 (29.2)	69 (31.9)	10 (18.2)	**0.045**
Malignancy	78 (28.8)	55 (25.5)	23 (41.8)	**0.017**
Cerebrovascular disease	59 (21.8)	48 (22.2)	11 (20.0)	0.721
Transplantation	16 (5.9)	11 (5.1)	5 (9.1)	0.332
Immunosuppressant use	37 (13.7)	28 (13.0)	9 (16.4)	0.512
**Clinical severity**				
Severe sepsis or septic shock	136 (50.2)	115 (53.2)	21 (38.2)	**0.046**
Pitt score, median (IQR)	4.00 (2.00–6.00)	5.00 (2.00–7.00)	2.00 (1.00–4.00)	** < 0.001**
Inappropriate empirical antibiotics	191 (70.5)	162 (75.0)	29 (52.7)	**0.001**
All-cause 30-day mortality	135 (51.1)	122 (57.5)	13 (25.0)	** < 0.001**
Treatment failure	150 (55.4)	131 (60.6)	19 (34.5)	**0.001**

SD, standard deviation; ICU, intensive care unit; CRBSI, catheter-related bloodstream infection; WIC, weighted index of comorbidity; IQR, interquartile range.

Significant values are in [bold].

### Antibiotic susceptibility of carbapenem-resistant isolates

Antibiotic susceptibility of carbapenem-resistant isolates is described in Table 2. Susceptibility test results were not available for all carbapenem-resistant isolates. The percentages of non-susceptible isolates differed significantly between the CR-Ab and CR-Pa groups for all antibiotics except for colistin and trimethoprim/sulfamethoxazole. The percentages of non-susceptible isolates, except for tigecycline and minocycline, were significantly higher in the CR-Ab group than in the CR-Pa group. Of the 55 CR-Pa cases, only 3 (5.5%) cases were susceptible to colistin only, and resistant to pan-drugs.

**Table 2:** Antibiotic susceptibility of carbapenem-resistant *Acinetobacter baumannii* (CR-Ab) and carbapenem-resistant *Pseudomonas aeruginosa* (CR-Pa) isolates.

Non-susceptible (I + R) isolate No. (%)	Total^a^ (n = 271)	CR-Ab (n = 216)	CR-Pa (n = 55)	*P*
Ceftazidime	240/268 (89.6)	209 (97.2)	31 (58.5)	** < 0.001**
Cefepime	242/265 (91.3)	211 (98.6)	31 (60.8)	** < 0.001**
Ciprofloxacin	239/269 (88.8)	210 (97.2)	29 (54.7)	** < 0.001**
Amikacin	66/130 (50.8)	58 (70.7)	8 (16.7)	** < 0.001**
Gentamicin	175/245 (71.4)	161 (77.8)	14 (36.8)	** < 0.001**
Tobramycin	45/88 (51.1)	40 (74.1)	5 (14.7)	** < 0.001**
Piperacillin/tazobactam	225/243 (92.6)	191 (99.0)	34 (68.0)	** < 0.001**
Trimethoprim/sulfamethoxazole	145/174 (83.3)	134 (82.2)	11 (100.0)	0.215
Imipenem	252/260 (96.9)	211 (99.1)	41 (87.2)	**0.001**
Meropenem	254/261 (97.3)	213 (99.5)	41 (87.2)	** < 0.001**
Tigecycline	24/118 (20.3)	16 (14.5)	8 (100.0)	** < 0.001**
Colistin	4/217 (1.8)	3 (1.7)	1 (2.7)	0.529
Minocycline	47/174 (27.0)	38 (23.2)	9 (90.0)	** < 0.001**

I, intermediate; R, resistant; ^a^Not all isolate susceptibility test results were available, and the results are presented as non-susceptible isolate number/available total (%).

Significant values are in [bold].

### Risk factors for treatment failure

The results of univariate analysis of risk factors for treatment failure in patients with Ab bacteraemia are shown in Supplementary Table S3. We stratified patient data according to carbapenem resistance and the appropriateness of empirical antibiotic therapy in the multivariate analyses of risk factors for treatment failure because of the significant interaction between these factors.

The risk factors for treatment failure in patients with Ab bacteraemia, stratified according to the appropriateness of empirical antibiotic therapy, are shown in Table 3. While severe sepsis or septic shock and high Pitt bacteraemia score were independent risk factors for treatment failure in the inappropriate empirical antibiotics group, pneumonia, and Charlson’s weighted index of comorbidity (WIC) ≥ 3 were independent risk factors for treatment failure in the appropriate empirical antibiotics group.

**Table 3:** Multivariate analysis of risk factors for treatment failure in patients with *Acinetobacter baumannii* bacteraemia, according to the appropriateness of empirical therapy.

Risk factors	Inappropriate empirical antibiotics (n = 179)		Appropriate empirical antibiotics (n = 125)	
	aOR (95% CI)	*P*	aOR (95% CI)	*P*
Male	–	–	2.36 (0.89–6.23)	0.084
ICU stay at bacteraemia onset	–	–	1.23 (0.36–4.25)	0.746
Healthcare-associated infection	5.14 (0.43–61.37)	0.196	–	–
**Site of infection**				
Primary bacteraemia	–	–	1.50 (0.47–4.78)	0.489
CRBSI	2.35 (0.98–5.64)	0.055	–	–
Pneumonia	–	–	10.56 (2.68–41.57)	**0.001**
**Underlying medical condition**				
Charlson’s WIC ≥ 3	–	–	4.66 (1.07–20.36)	**0.041**
Heart disease	2.72 (0.66–11.31)	0.168	–	–
Lung disease	–	–	4.32 (0.65–28.67)	0.130
**Clinical severity**				
Severe sepsis or septic shock	22.70 (9.48–54.39)	** < 0.001**	1.67 (0.60–4.66)	0.330
Pitt score	1.58 (1.36–1.83)	** < 0.001**	1.15 (0.94–1.41)	0.169
Carbapenem resistance	6.17 (1.13–33.75)	**0.036**	4.15 (1.16–14.84)	**0.029**

aOR, adjusted odds ratio; CI, confidence interval; ICU, intensive care unit; CRBSI, catheter related bloodstream infection; WIC, weighted index of comorbidity; –, the variable was not included in the multivariate analysis model, because it was not significant in univariate analysis (*P* > 0.10).

Significant values are in [bold].

The results of multivariate analysis of risk factors for treatment failure in patients with Ab bacteraemia according to carbapenem susceptibility are shown in Supplementary Table S4. In the CR-Ab group, severe sepsis or septic shock (adjusted odds ratio [aOR]: 9.70, 95% confidence interval [CI]: 5.02–18.76, *P* < 0.001) and high Pitt bacteraemia score (aOR: 1.44, 95% CI: 1.27–1.63, *P* < 0.001) were significantly associated with treatment failure.

The results of univariate analysis of risk factors for treatment failure in patients with Pa bacteraemia are listed in Supplementary Table S5. The risk factors for treatment failure in patients with Pa bacteraemia according to the appropriateness of empirical antibiotic therapy are shown in Table 4. HAI, severe sepsis or septic shock, and high Pitt score were independent risk factors for treatment failure in both the inappropriate and appropriate empirical antibiotics groups. Urinary tract infection was associated with a lower risk of treatment failure in the appropriate antibiotics group. Supplementary Table S6 shows the results of multivariate analysis of risk factors for treatment failure in patients with Pa bacteraemia according to carbapenem susceptibility. In the CR-Pa group, HAI (aOR: 25.03, 95% CI: 2.15–291.99, *P* = 0.010), use of immunosuppressant (aOR: 32.08, 95% CI: 1.58–652.62, *P* = 0.024), and high Pitt bacteraemia score (aOR: 1.47, 95% CI: 1.05–2.07, *P* = 0.025) were significantly associated with treatment failure.

**Table 4:** Multivariate analysis of risk factors for treatment failure in patients with *Pseudomonas aeruginosa* bacteraemia, according to the appropriateness of empirical therapy.

Risk factors	Inappropriate empirical antibiotics (n = 78)		Appropriate empirical antibiotics (n = 163)	
	aOR (95% CI)	*P*	aOR (95% CI)	*P*
Male	–	–	3.61 (1.23–10.65)	**0.020**
ICU stay at bacteraemia onset	–	–	1.32 (0.41–4.29)	0.645
Hospital-acquired infection	4.57 (1.34–15.54)	**0.015**	2.88 (1.09–7.65)	**0.033**
**Site of infection**				
Primary bacteraemia	–	–	2.79 (0.94–8.25)	0.064
Pneumonia	4.61 (0.68–31.20)	0.117	3.82 (1.09–13.32)	**0.036**
Urinary tract infection	–	–	0.13 (0.02–0.79)	**0.027**
**Underlying medical condition**				
Malignancy	2.09 (0.55–7.91)	0.276	1.81 (0.66–4.96)	0.251
Immunosuppressant use	3.31 (0.78–14.11)	0.105	1.58 (0.54–4.63)	0.406
**Clinical severity**				
Severe sepsis or septic shock	5.43 (1.42–20.76)	**0.013**	10.33 (3.56–29.94)	** < 0.001**
Pitt score	1.59 (1.21–2.10)	**0.001**	1.59 (1.27–1.99)	** < 0.001**
Carbapenem resistance	0.91 (0.27–3.03)	0.879	1.76 (0.45–6.87)	0.418

aOR, adjusted odds ratio; CI, confidence interval; ICU, intensive care unit.

Significant values are in [bold].

### Carbapenem resistance and treatment failure

In Ab bacteraemia, carbapenem resistance was independently associated with treatment failure in both the inappropriate (aOR: 6.17; 95% CI: 1.13–33.75; *P* = 0.036) and appropriate (aOR: 4.15; 95% CI: 1.16–14.84; *P* = 0.029; Table 3) empirical antibiotics groups. In Pa bacteraemia, carbapenem resistance was not significantly associated with treatment failure in both the inappropriate (aOR: 0.91, 95% CI: 0.27–3.03, *P* = 0.879) and appropriate (aOR: 1.76, 95% CI: 0.45–6.87, *P* = 0.418; Table 4) empirical antibiotics groups.

## Discussion

Resistance to carbapenem is an important concern in both Ab and Pa bacteraemia. Here, we found different clinical characteristics and impacts of carbapenem resistance on the outcomes of Ab and Pa bacteraemia.

Carbapenem resistance was an independent risk factor for treatment failure in Ab bacteraemia regardless of the appropriateness of empirical antibiotics. This finding is consistent with those of previous studies, which found that carbapenem resistance was significantly associated with mortality in Ab bacteraemia^7,8^. A systematic review found that carbapenem resistance may increase the risk of mortality in patients with Ab infection^9^. In contrast, we found that carbapenem resistance was not an independent risk factor for treatment failure in Pa bacteraemia regardless of the appropriateness of empirical antibiotics. This finding is in agreement with that of a previous study showing that carbapenem resistance was not an independent risk factor for mortality in Pa bacteraemia^10^. This difference in the impact of carbapenem resistance on Ab and Pa may be due to available treatment options, different sites of infection, and differences in clinical severity. We found a difference in the antibiotic susceptibility profiles of CR-Ab and CR-Pa. The lower percentage of non-susceptible isolates to several antibiotics among CR-Pa isolates, suggests that there are more available treatment options for CR-Pa than for CR-Ab. Differences between CR-Ab and CR-Pa in the sites of infection and clinical severity might also contribute to the different impact of carbapenem resistance on Ab and Pa.

In this study, the antibiotic susceptibilities and clinical characteristics in patients with Ab and Pa bacteraemia differed. In Ab bacteraemia, 71.1% (216/304) of the cases were carbapenem resistant, while only 22.8% (55/241) of the cases in Pa bacteraemia were carbapenem resistant. The higher percentage of carbapenem resistance in Ab bacteraemia compared with Pa bacteraemia is consistent with the findings of a previous study, which found that the proportion of carbapenem resistance was 92.1% in Ab strains and the proportions of resistance were 19.5% to imipenem and 18.1% to meropenem in Pa strains^11^. In this study, CR-Ab was generally resistant to almost all antibiotics; however, CR-Pa was susceptible to some antibiotics, such as ciprofloxacin and amikacin. The difference in antibiotic susceptibility might explain the difference in the proportion of inappropriate empirical therapies between the groups. However, the results of multivariate analysis stratified according to the appropriateness of empirical antibiotic therapy showed that carbapenem resistance was an independent risk factor for treatment failure in Ab, even if the empirical therapy was appropriate. The finding that specific risk factors such as carbapenem resistance was associated with treatment failure despite appropriate empirical therapy suggests that the virulence of CR-Ab might have contributed to the clinical outcome. A previous study suggested that high bacterial cytotoxicity significantly affected mortality in Ab ventilator-associated pneumonia^12^.

Treatment failure was not significantly different between CR-Pa and CS-Pa, whereas it was significantly different between CR-Ab and CS-Ab. Moreover, a previous study found a significant difference in hospital mortality between CR-Ab and CS-Ab, but not between CR-Pa and CS-Pa^13^. The CR-Ab group had a significantly higher percentage of treatment failure than the CR-Pa group (60.6% vs. 34.5%). The difference in clinical severity and antibiotic susceptibility between the CR-Ab and CR-Pa groups might have contributed to the differences in treatment failure. Clinicians should consider the higher rates of treatment failure when carbapenem resistance is detected in Ab bacteraemia.

In this study, pneumonia, Charlson’s WIC ≥ 3, and carbapenem resistance were independent risk factors for treatment failure in the appropriate empirical antibiotics group with Ab bacteraemia. This suggests that the risk of treatment failure is high, even if appropriate empirical antibiotics were administered, if pneumonia is the source of bacteraemia or if the Ab is carbapenem resistant. One study identified carbapenem resistance, neutropenia, and prolonged intensive care unit stay as independent risk factors for mortality in Ab bacteraemia^7^. Another study showed that septic shock, carbapenem resistance, pneumonia, and inappropriate definite antimicrobial therapy were independent risk factors for 14-day mortality^14^.

Independent risk factors for treatment failure in Pa bacteraemia included HAI, severe sepsis or septic shock, and high Pitt bacteraemia score, regardless of the appropriateness of empirical antibiotics. One retrospective study found that septic shock and high Acute Physiologic Assessment and Chronic Health Evaluation (APACHE) II score were independent risk factors for 14-day mortality^10^. Another study found that recent hospitalisation, corticosteroid treatment, Charlson’s WIC, non-urinary source, and high Sequential Organ Failure Assessment (SOFA) score were significant risk factors for 30-day mortality in Pa bacteraemia^15^.

Additional analysis, stratified according to carbapenem resistance, revealed that inappropriate empirical antibiotics were independently associated with treatment failure in the CS-Pa group. Appropriate empirical antibiotics, including antipseudomonal coverage, may be important in the management of patients with risk of *Pseudomonas* infection. The independent risk factors for treatment failure in the CR-Pa group were HAI, use of immunosuppressants, and high Pitt bacteraemia score. This finding is consistent with that of a previous study reporting that high Pitt bacteraemia score was a predictor of mortality in CR-Pa bacteraemia^16^.

In both the CR-Ab and CS-Ab groups, inappropriate empirical antibiotic therapy was not an independent risk factor for treatment failure. Furthermore, a previous study found that 28-day mortality was not independently associated with appropriate empirical antimicrobial therapy in invasive *Acinetobacter* infection^17^. Although other studies have found that appropriate antimicrobial therapy decreased 14-day mortality^18,19^, these studies defined appropriate antimicrobial therapy as administration of antibiotics within 48 h after the onset of bacteraemia and did not consider the appropriateness of empirical antibiotics.

This study has several limitations. First, as an observational study, it is prone to bias and unmeasured confounding factors. Second, no microbiological investigation other than antibiotic susceptibility tests was conducted on the strains. As the effect of carbapenemase on clinical outcomes is not clear, further studies are required to determine the effect of the microbiological characteristics on the outcome in Ab and Pa. Lastly, while colistin was the only drug available in Korea for pandrug-resistant Ab and difficult-to-treat Pa infection at the time of the study, several new drugs, such as ceftolozane-tazobactam, ceftazidime-avibactam, imipenem-relebactam, and cefiderocol, were available in other countries^2,20^. This difference in availability of drugs may have contributed to the differences in prognosis.

In conclusion, this study identified clinical characteristics and risk factors associated with treatment failure in Ab and Pa bacteraemia. Carbapenem resistance was associated with increased risk of treatment failure in Ab but not in Pa bacteraemia. Because carbapenem resistance is significantly associated with treatment failure in the appropriate empirical antibiotics group with Ab, this study suggests that appropriate empirical antibiotics may not improve the clinical outcomes in CR-Ab. Hence, we suggest that it would be important to reduce the occurrence and spread of CR-Ab through infection control in high-risk groups such as patients in intensive care units. In addition, different treatment approaches might be needed for Ab and Pa.

## Methods

### Patient population and clinical data

All patients with Ab or Pa bacteraemia were identified prospectively from clinical microbiology laboratories in 10 hospitals over a 1-year period between 1 September 2017 and 31 August 2018. Antimicrobial susceptibility testing of isolates from pure cultures was performed using the Vitek®2 (bioMérieux, France), MicroScan (Beckman Coulter, Inc., Atlanta, GA) and Phoenix (BD Diagnostics, Franklin Lakes, NJ). We prospectively collected patients’ demographic and microbiological data, underlying medical conditions, source of bacteraemia, clinical severity, antibiotic therapy, and clinical outcomes. This study was approved by the Seoul National University Bundang Hospital Institutional Review Board (No. B-1804–463-105) and the institutional review boards of each participating hospital. The study was conducted in accordance with the principles outlined in the 1964 Declaration of Helsinki and its later amendments. The need for informed consent was waived by the Seoul National University Bundang Hospital Institutional Review Board.

## Definitions

Mixed bacteraemia was defined as the isolation of other organisms except for common skin flora, such as coagulase-negative staphylococci^21^, from the first blood culture that yielded Pa or Ab. Bacteraemia detected in patients who have been hospitalised for over 48 h was classified as a HAI. Healthcare-associated infection was defined as fulfilling at least one of the following criteria: (1) hospitalisation for 2 or more days within the previous 12 months; (2) residence in a nursing home within the previous 12 months; (3) treatment with parenteral antibiotics within the previous 30 days; and (4) renal replacement therapy within the previous 30 days. Use of immunosuppressants was defined as the use of any anticancer chemotherapy, or corticosteroids in a dose of more than 20 mg of prednisolone equivalent over 1 week, or any other immunosuppressive agent within the previous 30 days.

Severity of illness was assessed using the Pitt bacteraemia score^22^, and the severity of the underlying disease was assessed using Charlson’s WIC^23^. Empirical antimicrobial therapy was defined as initial treatment used more than 24 h after the sampling of the first positive blood culture. Antibiotic therapy was considered inappropriate if the isolate was not susceptible to the treatment regimen in vitro.

Treatment failure was defined as fulfilling any of the following events: (1) death from any cause within 30 days; (2) persistent bacteraemia (defined as positive blood cultures after ≥ 7 days of appropriate antimicrobial therapy); (3) recurrent bacteraemia within 30 days after the discontinuation of appropriate antimicrobial therapy^24^.

### Data management and statistical analysis

The chi-squared test or Fisher’s exact test was used to compare categorical variables, and Student’s t-test or Mann–Whitney U test was used to compare continuous variables. A stepwise multiple logistic regression was used to identify independent risk factors for treatment failure. Risk factors with a *P* value of < 0.10 in univariate analysis and other variables of clinical significance were included in multivariate analysis. To avoid multicollinearity, severe sepsis or septic shock and Pitt bacteraemia score were analysed in a separate multivariate model. All underlying diseases and Charlson’s WIC were analysed in a separate multivariate model for the same reason. *P* values of < 0.05 were considered statistically significant. The Hosmer–Lemeshow test was performed to assess the goodness of fit for logistic regression models. All statistical analyses were performed using IBM SPSS Statistics for Windows, version 26.0 (IBM Corp., Armonk, NY, USA).

## Supplementary Information

Supplementary Information.

## Data Availability

The data that support the findings of this study are available from the corresponding author, KHS, upon reasonable request.

## References

[1] CDC. Antibiotic Resistance Threats in the United States, 2019. *Atlanta, GA: U.S. Department of Health and Human Services, CDC* (2019).

[2] Doi Y (2019). Treatment options for carbapenem-resistant gram-negative bacterial infections. Clin. Infect. Dis..

[3] Logan LK, Weinstein RA (2017). The epidemiology of carbapenem-resistant enterobacteriaceae: The impact and evolution of a global menace. J. Infect Dis..

[4] Zilberberg MD, Nathanson BH, Sulham K, Fan W, Shorr AF (2017). Carbapenem resistance, inappropriate empiric treatment and outcomes among patients hospitalized with Enterobacteriaceae urinary tract infection, pneumonia and sepsis. BMC Infect. Dis..

[5] Durante-Mangoni E, Andini R, Zampino R (2019). Management of carbapenem-resistant Enterobacteriaceae infections. Clin. Microbiol. Infect..

[6] Tomczyk S (2019). Control of carbapenem-resistant enterobacteriaceae, acinetobacter baumannii, and pseudomonas aeruginosa in healthcare facilities: A systematic review and reanalysis of quasi-experimental studies. Clin. Infect. Dis..

[7] Choe YJ, Lee HJ, Choi EH (2019). Risk factors for mortality in children with acinetobacter baumannii bacteremia in South Korea: The role of carbapenem resistance. Microb. Drug Resist..

[8] Park SY (2013). Risk factors for mortality in patients with Acinetobacter baumannii bacteremia. Infect. Chemother..

[9] Lemos EV (2014). Carbapenem resistance and mortality in patients with Acinetobacter baumannii infection: Systematic review and meta-analysis. Clin. Microbiol. Infect..

[10] Kim YJ (2014). Risk factors for mortality in patients with Pseudomonas aeruginosa bacteremia; retrospective study of impact of combination antimicrobial therapy. BMC Infect. Dis..

[11] Liu C (2019). Antimicrobial resistance in South Korea: A report from the Korean global antimicrobial resistance surveillance system (Kor-GLASS) for 2017. J. Infect. Chemother..

[12] Ju MH (2018). Risk factors for mortality in ICU patients with Acinetobacter baumannii ventilator-associated pneumonia: Impact of bacterial cytotoxicity. J. Thorac. Dis..

[13] Zhen X, Stalsby Lundborg C, Sun X, Gu S, Dong H (2020). Clinical and economic burden of carbapenem-resistant infection or colonization caused by klebsiella pneumoniae, pseudomonas aeruginosa, Acinetobacter baumannii: A multicenter study in China. Antibiotics (Basel).

[14] Kim YJ (2012). Risk factors for mortality in patients with carbapenem-resistant Acinetobacter baumannii bacteremia: Impact of appropriate antimicrobial therapy. J. Korean Med. Sci..

[15] Babich T (2020). Risk factors for mortality among patients with Pseudomonas aeruginosa bacteraemia: A retrospective multicentre study. Int. J. Antimicrob. Agents.

[16] Buehrle DJ (2017). Carbapenem-resistant pseudomonas aeruginosa bacteremia: Risk factors for mortality and microbiologic treatment failure. Antimicrob. Agents Chemother..

[17] Kiyasu Y, Hitomi S, Funayama Y, Saito K, Ishikawa H (2020). Characteristics of invasive Acinetobacter infection: A multicenter investigation with molecular identification of causative organisms. J. Infect. Chemother..

[18] Lee YT (2012). Impact of appropriate antimicrobial therapy on mortality associated with Acinetobacter baumannii bacteremia: Relation to severity of infection. Clin. Infect. Dis..

[19] Kang FY (2020). Influence of severity of infection on the effect of appropriate antimicrobial therapy for Acinetobacter baumannii bacteremic pneumonia. Antimicrob. Resist. Infect. Control.

[20] Tamma, P. D. *et al.* Infectious diseases society of America guidance on the treatment of extended-spectrum beta-lactamase producing enterobacterales (ESBL-E), carbapenem-resistant enterobacterales (CRE), and Pseudomonas aeruginosa with Difficult-to-Treat Resistance (DTR-P. aeruginosa). *Clin. Infect. Dis.* **72**, 1109–1116, 10.1093/cid/ciab295 (2021). 10.1093/cid/ciab295 33830222

[21] Hall KK, Lyman JA (2006). Updated review of blood culture contamination. Clin. Microbiol. Rev..

[22] Paterson DL (2004). International prospective study of Klebsiella pneumoniae bacteremia: implications of extended-spectrum beta-lactamase production in nosocomial Infections. Ann. Intern. Med..

[23] Charlson ME, Pompei P, Ales KL, MacKenzie CR (1987). A new method of classifying prognostic comorbidity in longitudinal studies: Development and validation. J. Chronic Dis..

[24] Chen SY (2012). Methicillin-resistant Staphylococcus aureus (MRSA) staphylococcal cassette chromosome mec genotype effects outcomes of patients with healthcare-associated MRSA bacteremia independently of vancomycin minimum inhibitory concentration. Clin. Infect. Dis..

