# Supplementary material for: Different clinical characteristics and impact of carbapenem-resistance on outcomes between Acinetobacter baumannii and Pseudomonas aeruginosa bacteraemia: a prospective observational study
Source: Sci Rep. 2022 May 20;12:8527. doi: 10.1038/s41598-022-12482-0 (PMC9123196; doi:10.1038/s41598-022-12482-0)
Supplement: Supplementary file 1 — Supplementary Information. [file 41598_2022_12482_MOESM1_ESM.docx]

Supplementary Table S1. Clinical characteristics of patients with carbapenem-resistant *Acinetobacter baumannii* (CR-Ab) and carbapenem-susceptible *A. baumannii* (CS-Ab) bacteraemia

| Variables | Total  (n=304) | CR-Ab  (n=216) | CS-Ab  (n=88) | *P* |
| --- | --- | --- | --- | --- |
| Age, mean (±SD) | 65.0 (±17.6) | 66.0 (±17.2) | 62.6 (±18.3) | 0.123 |
| Male | 190 (62.5) | 143 (66.2) | 47 (53.4) | **0.037** |
| ICU stay  at bacteraemia onset | 141 (46.4) | 127 (58.8) | 14 (15.9) | **<0.001** |
| Hospital-acquired infection | 237 (78.0) | 184 (85.2) | 53 (60.2) | **<0.001** |
| Healthcare-associated infection | 282 (92.8) | 211 (97.7) | 71 (80.7) | **<0.001** |
| Mixed bacteraemia | 28 (9.2) | 18 (8.3) | 10 (11.4) | 0.407 |
| **Site of infection** | | | | |
| Primary bacteraemia | 99 (32.6) | 58 (26.9) | 41 (46.6) | **0.001** |
| CRBSI | 116 (38.2) | 91 (42.1) | 25 (28.4) | **0.026** |
| Pneumonia | 86 (28.3) | 78 (36.1) | 8 (9.1) | **<0.001** |
| Urinary tract infection | 58 (19.1) | 45 (20.8) | 13 (14.8) | 0.223 |
| Intra-abdominal infection | 26 (8.6) | 16 (7.4) | 10 (11.4) | 0.263 |
| **Underlying medical condition** | | | | |
| Charlson’s WIC ≥ 3 | 239 (78.6) | 172 (79.6) | 67 (76.1) | 0.500 |
| Heart disease | 31 (10.2) | 24 (11.1) | 7 (8.0) | 0.409 |
| Lung disease | 30 (9.9) | 26 (12.0) | 4 (4.5) | **0.047** |
| Chronic kidney disease | 55 (18.1) | 43 (19.9) | 12 (13.6) | 0.198 |
| Liver disease | 60 (19.7) | 42 (19.4) | 18 (20.5) | 0.841 |
| Diabetes mellitus | 91 (29.9) | 69 (31.9) | 22 (25.0) | 0.231 |
| Malignancy | 86 (28.3) | 55 (25.5) | 31 (35.2) | 0.086 |
| Cerebrovascular disease | 69 (22.7) | 48 (22.2) | 21 (23.9) | 0.757 |
| Transplantation | 11 (3.6) | 11 (5.1) | 0 (0.0) | **0.038** |
| Immunosuppressant use | 46 (15.1) | 28 (13.0) | 18 (20.5) | 0.098 |
| **Clinical severity** | | | | |
| Severe sepsis or septic shock | 134 (44.1) | 115 (53.2) | 19 (21.6) | **<0.001** |
| Pitt score, median (IQR) | 4.00 (1.00–6.00) | 5.00 (2.00–7.00) | 1.00 (0.00–3.00) | **<0.001** |
| Inappropriate empirical antibiotics | 179 (58.9) | 162 (75.0) | 17 (19.3) | **<0.001** |
| All-cause 30-day mortality | 135 (45.6) | 122 (57.5) | 13 (15.5) | **<0.001** |
| Treatment failure | 145 (47.7) | 131 (60.6) | 14 (15.9) | **<0.001** |

SD, standard deviation; ICU, intensive care unit; CRBSI, catheter-related bloodstream infection; WIC, weighted index of comorbidity; IQR, interquartile range.

Supplementary Table S2. Clinical characteristics of patients with carbapenem-resistant *Pseudomonas aeruginosa* (CR-Pa) and carbapenem-susceptible *P. aeruginosa* (CS-Pa) bacteraemia

| Variables | Total  (n=241) | CR-Pa  (n=55) | CS-Pa  (n=186) | *P* |
| --- | --- | --- | --- | --- |
| Age, mean (±SD) | 66.4 (±15.3) | 64.1 (±15.4) | 67.1 (±15.3) | 0.213 |
| Male | 150 (62.2) | 34 (61.8) | 116 (62.4) | 0.941 |
| ICU stay  at bacteraemia onset | 44 (18.3) | 12 (21.8) | 32 (17.2) | 0.436 |
| Hospital-acquired infection | 118 (49.0) | 34 (61.8) | 84 (45.2) | **0.030** |
| Healthcare-associated infection | 211 (87.6) | 49 (89.1) | 162 (87.1) | 0.694 |
| Mixed bacteraemia | 35 (14.5) | 11 (20.0) | 24 (12.9) | 0.189 |
| **Site of infection** | | | | |
| Primary bacteraemia | 77 (32.0) | 18 (32.7) | 59 (31.7) | 0.888 |
| CRBSI | 40 (16.6) | 12 (21.8) | 28 (15.1) | 0.236 |
| Pneumonia | 34 (14.1) | 4 (7.3) | 30 (16.1) | 0.097 |
| Urinary tract infection | 59 (24.5) | 10 (18.2) | 49 (26.3) | 0.216 |
| Intra-abdominal infection | 33 (13.7) | 10 (18.2) | 23 (12.4) | 0.270 |
| **Underlying medical condition** | | | | |
| Charlson’s WIC ≥ 3 | 192 (79.7) | 44 (80.0) | 148 (79.6) | 0.944 |
| Heart disease | 11 (4.6) | 4 (7.3) | 7 (3.8) | 0.279 |
| Lung disease | 8 (3.3) | 1 (1.8) | 7 (3.8) | 0.686 |
| Chronic kidney disease | 34 (14.1) | 8 (14.5) | 26 (14.0) | 0.915 |
| Liver disease | 40 (16.6) | 14 (25.5) | 26 (14.0) | **0.044** |
| Diabetes mellitus | 57 (23.7) | 10 (18.2) | 47 (25.3) | 0.277 |
| Malignancy | 112 (46.5) | 23 (41.8) | 89 (47.8) | 0.431 |
| Cerebrovascular disease | 36 (14.9) | 11 (20.0) | 25 (13.4) | 0.231 |
| Transplantation | 14 (5.8) | 5 (9.1) | 9 (4.8) | 0.321 |
| Immunosuppressant use | 68 (28.2) | 9 (16.4) | 59 (31.7) | **0.026** |
| **Clinical severity** | | | | |
| Severe sepsis or septic shock | 86 (35.7) | 21 (38.2) | 65 (34.9) | 0.660 |
| Pitt score, median (IQR) | 1.00 (0.00–3.00) | 2.00 (1.00–4.00) | 1.00 (0.00–3.00) | **0.045** |
| Inappropriate empirical antibiotics | 78 (32.4) | 29 (52.7) | 49 (26.3) | **<0.001** |
| All-cause 30-day mortality | 56 (25.0) | 13 (25.0) | 43 (25.0) | >0.999 |
| Treatment failure | 65 (27.0) | 19 (34.5) | 46 (24.7) | 0.150 |

SD, standard deviation; ICU, intensive care unit; CRBSI, catheter-related bloodstream infection; WIC, weighted index of comorbidity; IQR, interquartile range

Supplementary Table S3. Univariate analysis of risk factors for treatment failure in patients with *Acinetobacter baumannii* bacteraemia

| Risk factors | Treatment success  (n=159) | Treatment failure  (n=145) | OR (95% CI) | *P* |
| --- | --- | --- | --- | --- |
| Age, mean (±SD) | 63.0 (±18.3) | 67.1 (±16.5) | 1.01 (1.00–1.03) | **0.044** |
| Male | 93 (58.5) | 97 (66.9) | 1.43 (0.90–2.29) | 0.131 |
| ICU stay  at bacteraemia onset | 57 (35.8) | 84 (57.9) | 2.46 (1.55–3.91) | **<0.001** |
| Hospital-acquired infection | 116 (73.0) | 121 (83.4) | 1.87 (1.07–3.27) | **0.029** |
| Healthcare-associated infection | 140 (88.1) | 142 (97.9) | 6.42 (1.86–22.19) | **0.003** |
| Mixed bacteraemia | 19 (11.9) | 9 (6.2) | 0.49 (0.21–1.12) | 0.089 |
| **Site of infection** | | | | |
| Primary bacteraemia | 58 (36.5) | 41 (28.3) | 0.69 (0.42–1.12) | 0.128 |
| CRBSI | 56 (35.2) | 60 (41.4) | 1.30 (0.82–2.06) | 0.270 |
| Pneumonia | 29 (18.2) | 57 (39.3) | 2.90 (1.72–4.90) | **<0.001** |
| Urinary tract infection | 30 (18.9) | 28 (19.3) | 1.03 (0.58–1.82) | 0.922 |
| Intra-abdominal infection | 17 (10.7) | 9 (6.2) | 0.55 (0.24–1.28) | 0.167 |
| **Underlying medical condition** | | | | |
| Charlson’s WIC ≥ 3 | 119 (74.8) | 120 (82.8) | 1.61 (0.92–2.83) | 0.094 |
| Heart disease | 13 (8.2) | 18 (12.4) | 1.59 (0.75–3.38) | 0.226 |
| Lung disease | 11 (6.9) | 19 (13.1) | 2.03 (0.93–4.42) | 0.075 |
| Chronic kidney disease | 22 (13.8) | 33 (22.8) | 1.84 (1.01–3.33) | **0.045** |
| Liver disease | 29 (18.2) | 31 (21.4) | 1.22 (0.69–2.15) | 0.492 |
| Diabetes mellitus | 48 (30.2) | 43 (29.7) | 0.98 (0.60–1.59) | 0.919 |
| Malignancy | 47 (29.6) | 39 (26.9) | 0.88 (0.53–1.45) | 0.607 |
| Cerebrovascular disease | 41 (25.8) | 28 (19.3) | 0.69 (0.40–1.19) | 0.179 |
| Transplantation | 2 (1.3) | 9 (6.2) | 5.20 (1.10–24.46) | **0.037** |
| Immunosuppressant use | 21 (13.2) | 25 (17.2) | 1.37 (0.73–2.57) | 0.328 |
| **Clinical severity** | | | | |
| Severe sepsis or septic shock | 31 (19.5) | 103 (71.0) | 10.13 (5.95–17.23) | **<0.001** |
| Pitt score, median (IQR) | 1.00 (0.00–4.00) | 6.00 (4.00–8.00) | 1.53 (1.38–1.69) | **<0.001** |
| Inappropriate empirical antibiotics | 76 (47.8) | 103 (71.0) | 2.68 (1.67–4.31) | **<0.001** |
| Carbapenem resistance | 85 (53.5) | 131 (90.3) | 8.15 (4.33–15.34) | **<0.001** |

OR, odds ratio; CI, confidence interval; SD, standard deviation; ICU, intensive care unit; CRBSI, catheter-related bloodstream infection; WIC, weighted index of comorbidity; IQR, interquartile range

Supplementary Table S4. Multivariate analysis of risk factors for treatment failure in patients with *Acinetobacter baumannii* bacteraemia, according to carbapenem susceptibility

| Risk factors | CR-Ab (n=216) | | CS-Ab (n=88) | |
| --- | --- | --- | --- | --- |
|  | aOR (95% CI) | *P* | aOR (95% CI) | *P* |
| Age | - | - | 1.05 (0.99–1.12) | 0.104 |
| Healthcare-associated infection | 4.62 (0.35–60.73) | 0.245 | - | - |
| Mixed bacteraemia | - | - | - | - |
| **Site of infection** | | | | |
| Pneumonia | 1.64 (0.82–3.28) | 0.160 | 3.24 (0.30–34.84) | 0.332 |
| **Underlying medical condition** | | | | |
| Lung disease | - | - | 17.65 (0.80–387.39) | 0.069 |
| Chronic kidney disease | - | - | 4.23 (0.78–22.96) | 0.095 |
| Immunosuppressant use | 2.41 (0.81–7.19) | 0.114 | - | - |
| **Clinical severity** | | | | |
| Severe sepsis or septic shock | 9.70 (5.02–18.76) | **<0.001** | 2.44 (0.58–10.27) | 0.223 |
| Pitt score | 1.44 (1.27–1.63) | **<0.001** | 1.52 (1.13–2.05) | **0.006** |
| Inappropriate empirical antibiotics | 1.24 (0.59–2.59) | 0.577 | 0.05 (0.00–1.19) | 0.064 |

CR-Ab, carbapenem-resistant *Acinetobacter baumannii*; CS-Ab, carbapenem-susceptible *A. baumannii* bacteraemia; aOR, adjusted odds ratio; CI, confidence interval

Supplementary Table S5. Univariate analysis of risk factors for treatment failure in patients with *Pseudomonas aeruginosa* bacteraemia

| Risk factors | Treatment success  (n=176) | Treatment failure  (n=65) | OR (95% CI) | *P* |
| --- | --- | --- | --- | --- |
| Age, mean (±SD) | 66.3 (±15.6) | 66.6 (±14.7) | 1.00 (0.98–1.02) | 0.892 |
| Male | 104 (59.1) | 46 (70.8) | 1.68 (0.91–3.10) | 0.099 |
| ICU stay  at bacteraemia onset | 26 (14.8) | 18 (27.7) | 2.21 (1.11–4.38) | **0.023** |
| Hospital-acquired infection | 77 (43.8) | 41 (63.1) | 2.20 (1.22–3.94) | **0.008** |
| Healthcare-associated infection | 152 (86.4) | 59 (90.8) | 1.55 (0.60–3.99) | 0.361 |
| Mixed bacteraemia | 27 (15.3) | 8 (12.3) | 0.78 (0.33–1.81) | 0.554 |
| **Site of infection** | | | | |
| Primary bacteraemia | 50 (28.4) | 27 (41.5) | 1.79 (0.99–3.24) | 0.054 |
| CRBSI | 30 (17.0) | 10 (15.4) | 0.89 (0.41–1.93) | 0.759 |
| Pneumonia | 17 (9.7) | 17 (26.2) | 3.31 (1.57–6.98) | **0.002** |
| Urinary tract infection | 52 (29.5) | 7 (10.8) | 0.29 (0.12–0.67) | **0.004** |
| Intra-abdominal infection | 23 (13.1) | 10 (15.4) | 1.21 (0.54–2.70) | 0.643 |
| **Underlying medical condition** | | | | |
| Charlson’s WIC ≥ 3 | 136 (77.3) | 56 (86.2) | 1.83 (0.83–4.02) | 0.132 |
| Heart disease | 8 (4.5) | 3 (4.6) | 1.02 (0.26–3.95) | 0.982 |
| Lung disease | 5 (2.8) | 3 (4.6) | 1.66 (0.38–7.13) | 0.499 |
| Chronic kidney disease | 26 (14.8) | 8 (12.3) | 0.81 (0.35–1.89) | 0.626 |
| Liver disease | 27 (15.3) | 13 (20.0) | 1.38 (0.66–2.87) | 0.390 |
| Diabetes mellitus | 44 (25.0) | 13 (20.0) | 0.75 (0.37–1.51) | 0.419 |
| Malignancy | 72 (40.9) | 40 (61.5) | 2.31 (1.29–4.14) | **0.005** |
| Cerebrovascular disease | 29 (16.5) | 7 (10.8) | 0.61 (0.25–1.47) | 0.274 |
| Transplantation | 10 (5.7) | 4 (6.2) | 1.09 (0.33–3.60) | 0.889 |
| Immunosuppressant use | 40 (22.7) | 28 (43.1) | 2.57 (1.41–4.71) | **0.002** |
| **Clinical severity** | | | | |
| Severe sepsis or septic shock | 44 (25.0) | 42 (64.6) | 5.45 (2.97–10.11) | **<0.001** |
| Pitt score, median (IQR) | 1.00 (0.00–2.00) | 4.00 (1.00–7.00) | 1.46 (1.29–1.66) | **<0.001** |
| Inappropriate empirical antibiotics | 51 (29.0) | 27 (41.5) | 1.74 (0.96–3.15) | 0.066 |
| Carbapenem resistance | 36 (20.5) | 19 (29.2) | 1.61 (0.84–3.07) | 0.152 |

OR, odds ratio; CI, confidence interval; SD, standard deviation; ICU, intensive care unit; CRBSI, catheter-related bloodstream infection; WIC, weighted index of comorbidity; IQR, interquartile range

Supplementary Table S6. Multivariate analysis of risk factors for treatment failure in patients with *Pseudomonas aeruginosa* bacteraemia, according to carbapenem susceptibility

| Risk factors | CR-Pa (n=55) | | CS-Pa (n=186) | |
| --- | --- | --- | --- | --- |
|  | aOR (95% CI) | *P* | aOR (95% CI) | *P* |
| Male | - | **-** | 2.64 (1.07–6.49) | **0.034** |
| ICU stay  at bacteraemia onset | - | **-** | 2.99 (1.05–8.52) | **0.040** |
| Hospital-acquired infection | 25.03 (2.15–291.99) | **0.010** | - | **-** |
| **Site of infection** | | | | |
| Primary bacteraemia | 2.77 (0.53–14.37) | 0.225 | - | **-** |
| Pneumonia | - | **-** | 3.45 (1.25–9.49) | **0.017** |
| Urinary tract infection | - | **-** | 0.32 (0.10–0.97) | **0.044** |
| **Underlying medical condition** | | | | |
| Malignancy | 1.84 (0.37–9.14) | 0.458 | 1.68 (0.65–4.35) | 0.282 |
| Cerebrovascular disease | 0.07 (0.00–4.41) | 0.205 | - | **-** |
| Immunosuppressant use | 32.08 (1.58–652.62) | **0.024** | 2.04 (0.80–5.19) | 0.134 |
| **Clinical severity** | | | | |
| Severe sepsis or septic shock | 5.11 (0.92–28.20) | 0.062 | 6.10 (2.49–14.91) | **<0.001** |
| Pitt score | 1.47 (1.05–2.07) | **0.025** | 1.52 (1.24–1.86) | **<0.001** |
| Inappropriate empirical antibiotics | 1.38 (0.26–7.41) | 0.707 | 3.90 (1.47–10.33) | **0.006** |

CR-Pa, carbapenem-resistant *Pseudomonas aeruginosa*; CS-Pa, carbapenem-susceptible *P. aeruginosa* bacteraemia; aOR, adjusted odds ratio; CI, confidence interval; ICU, intensive care unit
